# THE CRUCIAL ROLE OF LEPTIN IN MEDIATING VISCERAL ADIPOSITY-ASSOCIATED AGING PROCESS

**DOI:** 10.1093/geroni/igad104.1748

**Published:** 2023-12-21

**Authors:** Shangang Zhao

**Affiliations:** UT Health San Antonio - Barshop Institute, San Antonio, Texas, United States

## Abstract

Visceral adiposity is a hallmark of aging and an important contributor to reduced healthspan and lifespan. The factors that mediate the detrimental effects of aging-associated visceral adiposity are poorly identified. Here, we have shown that hyperleptinemia, induced by a leptin transgenic cassette in adipose tissue, accelerates diet-induced obesity and glucose intolerance. In addition, hyperleptinemia promotes adipose tissue inflammation. Chronic culture of stromal vascular fraction (SVF) with leptin reduces the capacity of differentiation and increases inflammation. Moreover, chronic hyperleptinemia in fat depots accelerates the development senescent cells to establish a senescent-associated secretory phenotype (SASP), including increased local inflammation. This result indicates hyperleptinemia may be a “unidentified” driver for cellular senescence. Moreover, deletion of leptin in visceral fat depots significantly reduces visceral adiposity and senescent-associated secretory phenotype (SASP). These results highlight the crucial role of leptin in establishing SASP and targeting leptin may be a novel approach to alleviate aging-associated metabolic syndromes.

